# Supplementary material for: Depletion of aneuploid cells is shaped by cell-to-cell interactions
Source: Cell Genom. 2025 Jun 3;5(8):100894. doi: 10.1016/j.xgen.2025.100894 (PMC12366656; doi:10.1016/j.xgen.2025.100894)
Supplement: Document S1. Figures S1–S6 and Tables S1–S3 [file mmc1.pdf]

Cell Genomics, Volume *5*

Supplemental information

Depletion of aneuploid cells is shaped by cell-to-cell interactions

Elena Fusari, Mariana Muzzopappa, Juliette Gracia, and Marco Milán

Supplementary figures and tables

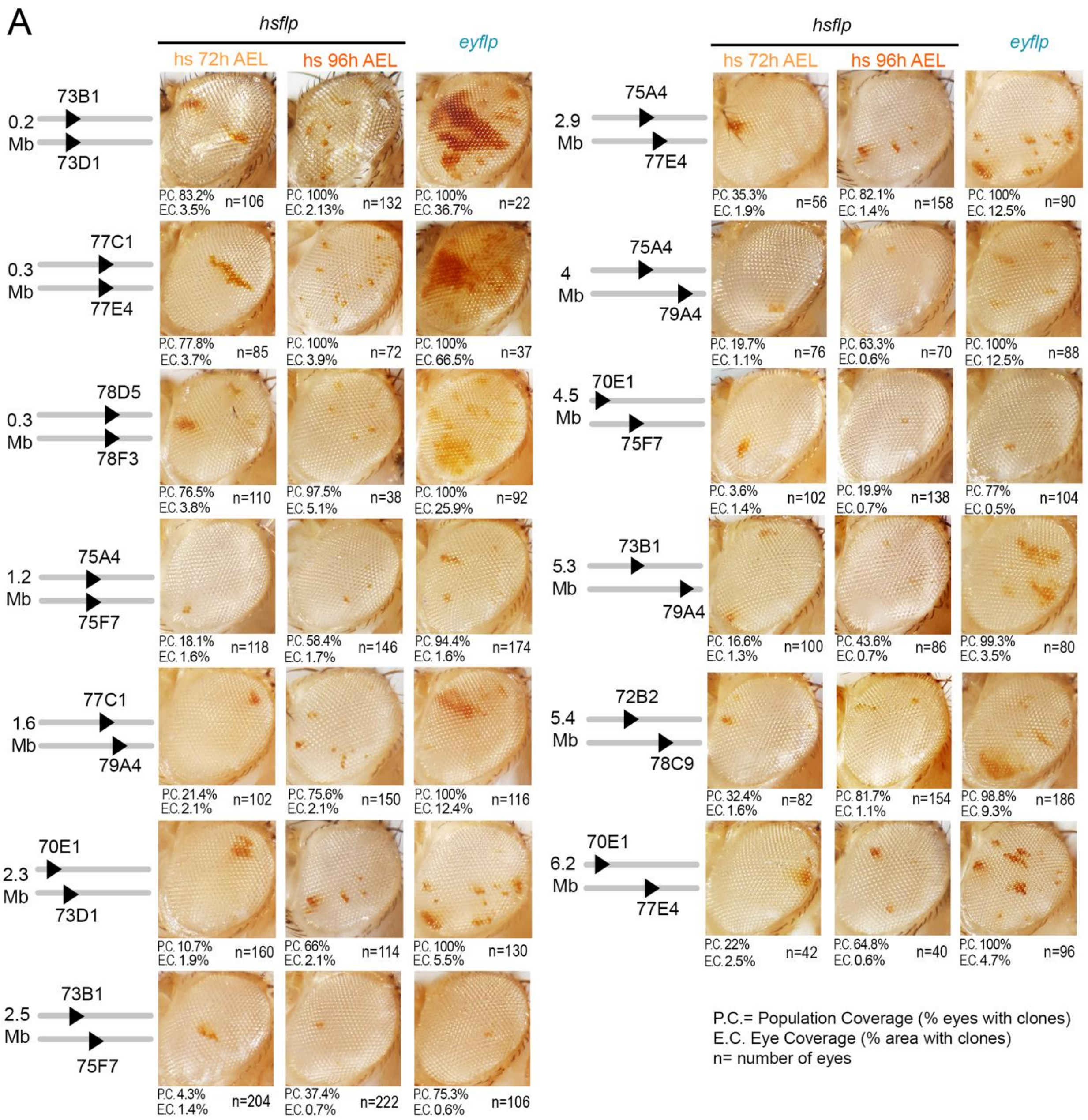

Figure S1. Recombination efficiency relies on genomic distance (related to Figure 1)

(A) Adult eyes with clones of cells labeled in red as a result of a recombination event between two RS-FRTs placed at the indicated genomic locations. Population and eye coverages (P.C. and E.C., respectively) and number of eyes scored for each FRT combination are shown.

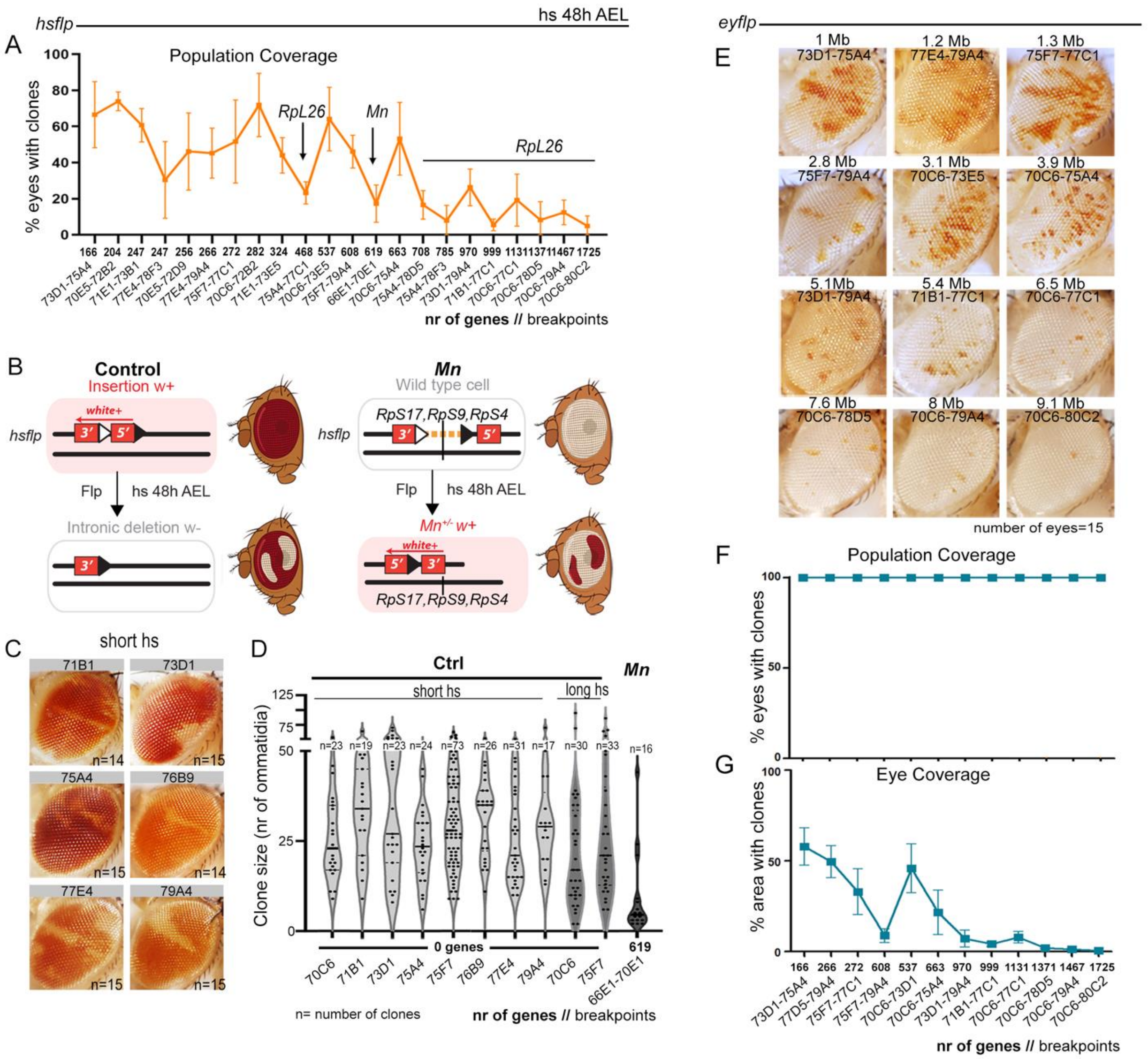

Figure S2. The effect of genomic distance on recombination efficiency (related to Figure 2)

(**A**, **F**, **G**) Plots representing the impact of the genomic distance (in number of genes) between the two FRTs on population coverage (% of eyes with clones, **A**, **F**) and eye coverage (% area with clones, **G**) of clones induced either acutely at 48h AEL (in orange, **A**) or chronically with the ey-FLP construct (in blue, **F**, **G**). Genomic location of each FRT combination is also indicated. (**B**) Drawings of clones of cells labeled in white (left) or red (right) as a result of a recombination event between two RS-FRTs located in the same

chromosome (in *cis*). Clones acting as controls delete the 5' intron of the *white* gene without affecting any gene (left), whereas clones inducing segmental monosomies and including *RpS17*, *RpS9* and *RpS4* (right) reconstitute the *white* gene. Recombination events are induced in founder cells during the growing period of the eye primordium and these cells proliferate and grow to give rise to clones visible in the adult eye. (**C**) Adult eyes with clones of cells labeled in white as a result of a recombination event within RS-FRTs placed at the indicated genomic locations. Clones in **A**, **C** and **D** were induced acutely at 48 h after egg laying (AEL). (**D**) Plot representing the size of control clones (in number of ommatidia) induced with the indicated RS-FRTs with short (light gray) and long (dark grey) heat-shock protocols (see Materials and Methods). Genomic location of RS-FRTs is indicated. Mean and SD are shown in **A**, **F**, **G**. Median is shown as a black line in **D**.

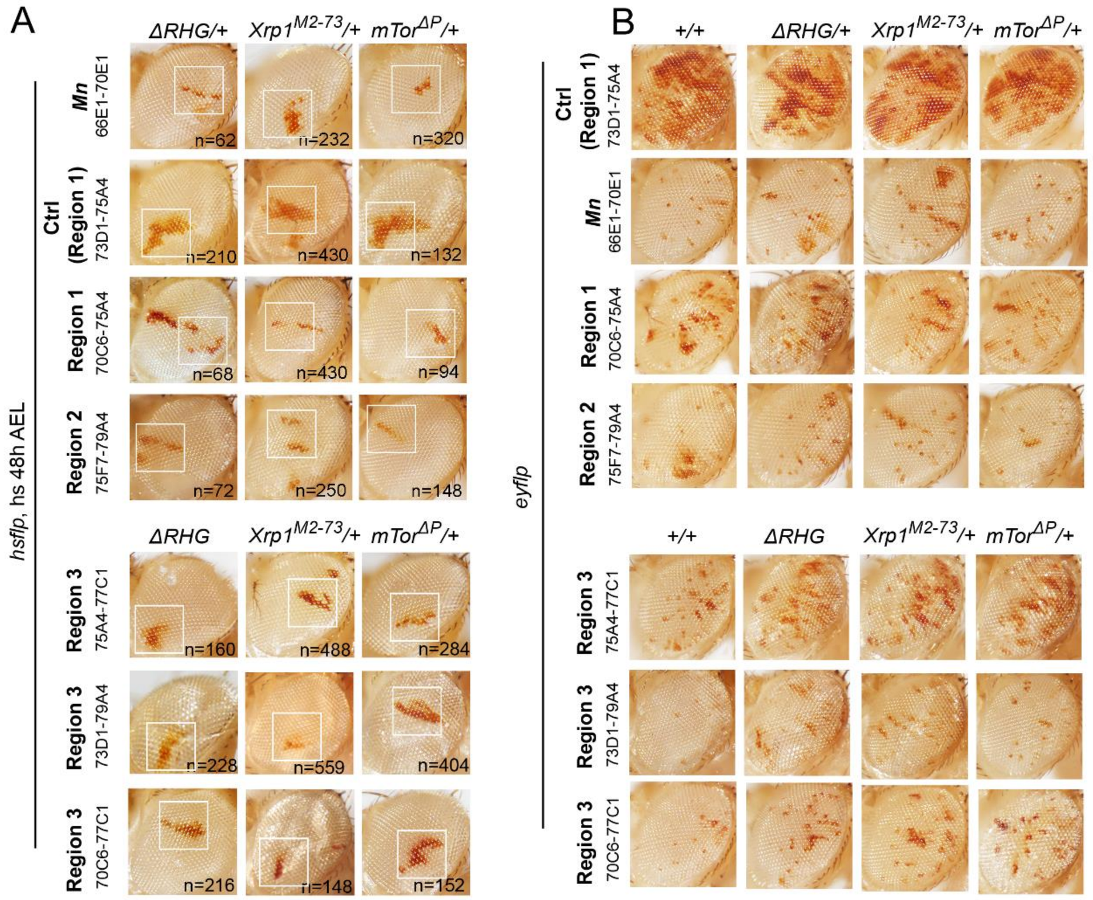

Figure S3. Cells carrying segmental monosomies show signs of out-competition (related to Figure 3)

(**A**, **B**) Adult eyes with clones of cells labeled in red, induced acutely at 48 h AEL (**A**) or chronically with *ey-FLP* (**B**) and bearing segmental monosomies spanning the indicated genomic locations. Clones were induced either in a wild-type background or in the indicated genetic backgrounds that compromise or block cell death, or half the doses of the *Xrp1* and *mTor* genes. High magnifications of the squared regions are shown in Figure 3A.

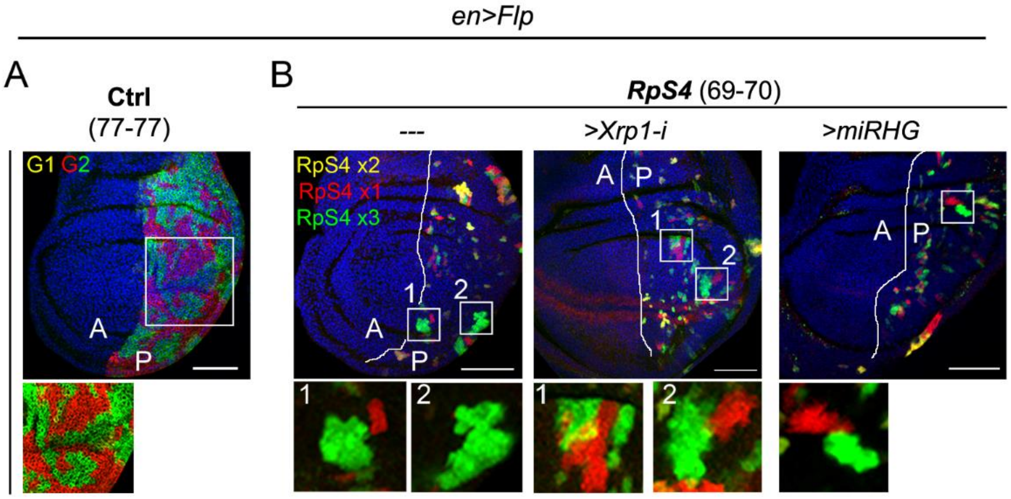

Figure S4. The TSG technique can detect *Minute*-driven cell competition (related to Figure 4)

(**A**, **B**) Wing primordia subjected to chronic expression of *FLP* under the control of the *en-gal4* driver. Due to the high recombination efficiency of FRTs located in the same genomic location, all control clones in **A** were either red or green due to the G1 products of recombination marked in yellow resolving into segmental monosomies and trisomies by further FRT-driven recombination in G2. In **B**, clones with one (red), two (yellow) and three (green) doses of the *RpS4* gene are observed. As indicated, tissues expressed an RNAi form of *Xrp1* or the *miRHG* transgene in the posterior (P) compartment. High magnification of the squared regions is shown in the lower panels. Scale bars, 50 µm.

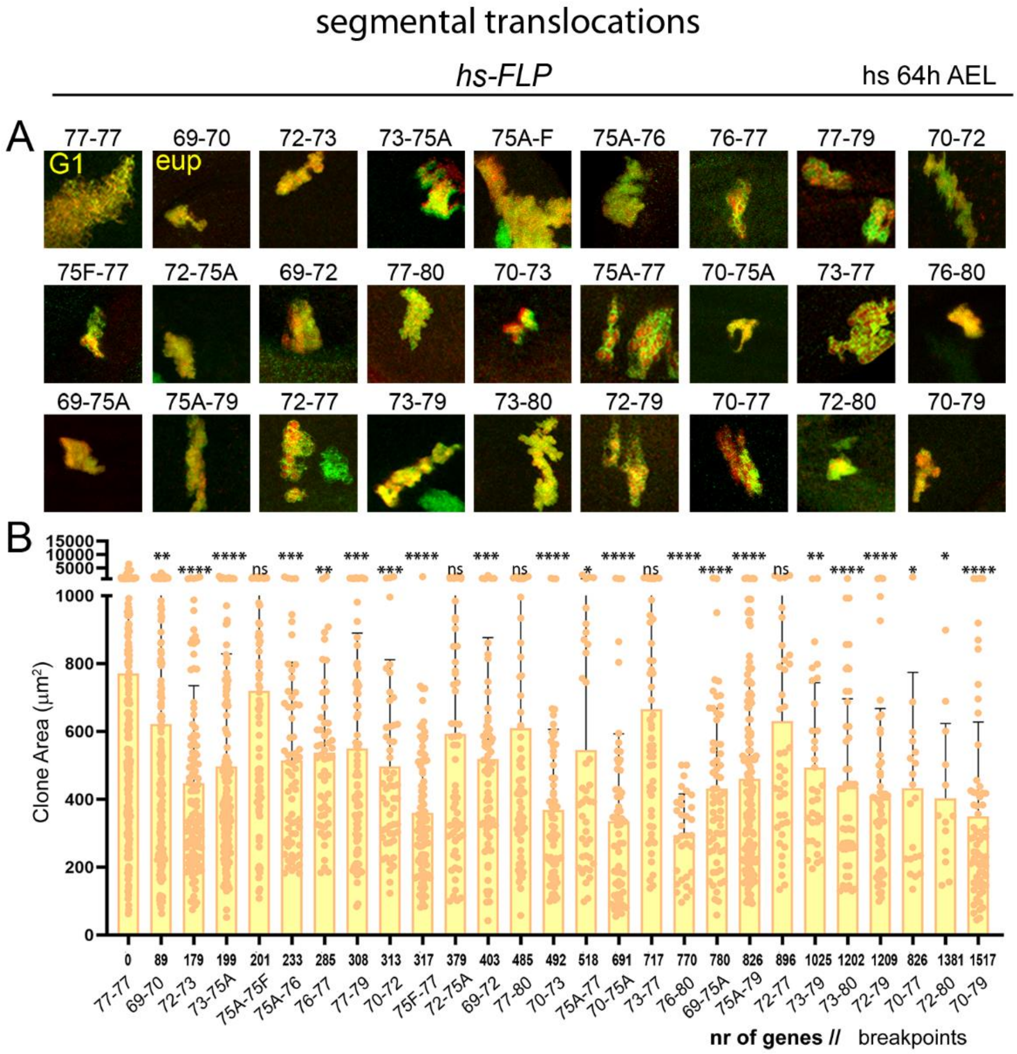

Figure S5. Effects of segmental translocation on clonal growth (related to Figure 5)

(**A**) Representative examples of clones of cells in wing primordia bearing segmental translocations spanning the indicated genomic locations and induced at the indicated developmental time. (**B**) Plot representing the impact of the size (in number of protein-encoding genes) of the segmental translocation on clone size (in μm^2). Genomic breakpoints of these translocations are indicated. Mean and SD are shown. Two-way ANOVA with Šidák correction for multiple comparisons test was performed. ns, not significant (p>0.05); * p ≤ 0.05; ** p ≤ 0.01; *** p≤ 0.001; and **** p ≤ 0.0001.

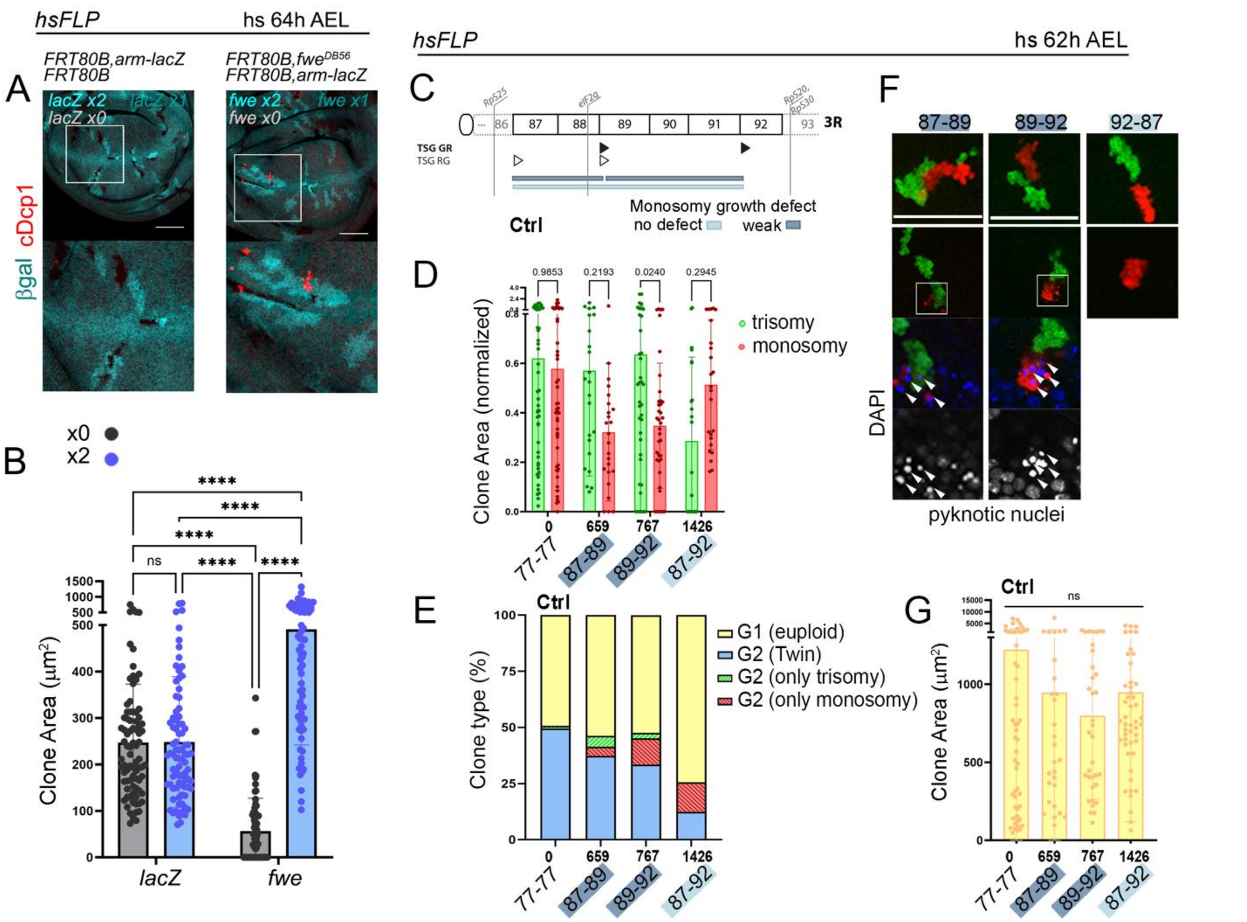

Figure S6. Cell interactions shape the behavior of aneuploid cells: cell competition and compensation (related to Figure 6)

(**A**) Wing disc with clones of wild type cells (left) or cells mutant for the *fwe* gene (right) marked by the absence of β-gal expression (cyan) and labeled to visualize cleaved Dcp1 (cDCp1, red). (**C**) Drawing of the right arm of the third chromosome and genomic coverage of segmental monosomies and trisomies produced. Genomic location of Ribosomal Protein (Rp)-encoding genes and *eIF2γ*, and the two types of TSG-FRTs (TSG-GR, black triangles, and TSG-RG, white triangles) used to simultaneously induce monosomic and trisomic cells are shown. Color code corresponds to the effect on growth of segmental monosomies. (**B**, **D**, **E**, **G**) Plots representing area (in μm^2, **B**, **G**, or normalized to the one of euploid cells, **D**) of control clones (grey in **B**, first data set in **D**), clones with different copies of the *fwe* gene (**B**), and clones bearing monosomies (red), trisomies (green) or translocations (yellow) spanning the indicated genomic locations and induced at the indicated developmental times (**D**), and distribution of the indicated types of clones (**E**). Mean and SD **(B, D**,

G) and average (**E**) are shown. Two-way ANOVA with Šidák correction for multiple comparisons test was performed in **B**, **D**, **G**. ns, not significant ($p>0.05$); * $p \leq 0.05$; ** $p \leq 0.01$; *** $p \leq 0.001$; and **** $p \leq 0.0001$. (**F**) Representative examples of clones of cells in wing primordia bearing segmental monosomies (red) or trisomies (green) spanning the indicated genomic locations and induced at the indicated developmental times. Arrowheads point to pyknotic nuclei labeled by DAPI (blue or white). Scale bars in **B**, **F**, 50 µm.

FRT type	Line ID	Origin	insertion	orientation respect to chr
Rs5(+)	126198	DGRC	70E1	+
Rs5(+)	126092	DGRC	72B2	+
Rs5(+)	125905	DGRC	73B1	+
Rs5(+)	126251	DGRC	73E5	+
Rs5(+)	126206	DGRC	75A4	+
Rs5(+)	125973	DGRC	77C1	+
Rs5(+)	125605	DGRC	78F3	+
Rs5(+)	125886	DGRC	78D5	+
Rs5(+)	126103	DGRC	79A2	+
Rs5(+)	126201	DGRC	79A4	+
Rs5(+)	123975	DGRC	80C2	+
Rs3(-)	124054	DGRC	70C6	+
Rs3(-)	123520	DGRC	70E5	+
Rs3(-)	123418	DGRC	71B1	+
Rs3(-)	123026	DGRC	71E1	+
Rs3(-)	123095	DGRC	73D1	+
Rs3(-)	124049	DGRC	75F7	+
Rs3(-)	124213	DGRC	77E4	+
Rs3(-)	123708	DGRC	78C9	+
Rs3(-)	124172	DGRC	78F3	+
Rs3(-)	124151	DGRC	79A4	+

Table S1. List of RS (Rearrangement Screening) FRTs used in this work (related to Figure 1)

RS-FRTs (RS3(-) or RS5(+)) used in this work were located in intergenic regions in the indicated genomic regions. Line ID and origin (DGRC, Drosophila Genomics Resource Center) is also indicated.

Genotype		Label	Pos RS3	Pos RS5	RS3 DGRC ID	RS5 DGRC ID	RS3 nt	RS5 nt	Diff(bp)	Mb	N genes
eyflp; 73-75	*hsflp; 73-75*	**73-75**	73D1	75A4	123095	126206	16800857	17850544	1049687	1,049687	166
eyflp; 70-72B2	*hsflp; 70-72B2*	**70-72B2**	70E5	72B2	123520	126092	14625700	15948261	1322561	1,322561	204
eyflp; 71-73B1	*hsflp; 71-73B1*	**71-73B1**	71E1	73B1	123026	125905	15525670	16605347	1079677	1,079677	247
eyflp; 77-78	*hsflp; 77-78*	**77-78**	77E4	78F3	124213	125605	20723348	21815069	1091721	1,091721	247
eyflp; 70E5-72D9	*hsflp; 70E5-72D9*	**70E5-72D9**	70E5	72D9	123520	125972	14625700	16157381	1531681	1,531681	256
eyflp; 77-79	*hsflp; 77-79*	**77-79**	77E4	79A4	124213	126201	20723348	21935345	1211997	1,211997	266
eyflp; 75-77	*hsflp; 75-77*	**75-77**	75F7	77C1	124049	125973	19094051	20394712	1300661	1,300661	272
eyflp; 70C6-72B2	*hsflp; 70C6-72B2*	**70C6-72B2**	70C6	72B2	124054	126092	13932268	15948261	2015993	2,015993	282
eyflp; 71-73E5	*hsflp; 71-73E5*	**71-73E5**	71E1	73E5	123026	126251	15525670	17042518	1516848	1,516848	324
eyflp; 75A4-77	*hsflp; 75A4-77*	**75A4-77**	75A4	77C1	124168	125973	17850477	20394712	2544235	2,544235	468
eyflp; 70-73	*hsflp; 70-73*	**70-73**	70C6	73E5	124054	126251	13932268	17042518	3110250	3,11025	537
eyflp; 75-79	*hsflp; 75-79*	**75-79**	75F7	79A4	124049	126201	19094051	21935345	2841294	2,841294	608
eyflp; 66-70	*hsflp; 66-70*	**66-70**	66E1	70E1	123215	126198	8820579	14530694	5710115	5,710115	619
eyflp; 70-75	*hsflp; 70-75*	**70-75**	70C6	75A4	124054	126206	13932268	17850544	3918276	3,918276	663
eyflp; 75A4-78D5	*hsflp; 75A4-78D5*	**75A4-78D5**	75A4	78D5	124168	125886	17850477	21526856	3676379	3,676379	708
eyflp; 75A4-78F3	*hsflp; 75A4-78F3*	**75A4-78F3**	75A4	78F3	124168	125605	17850477	21815069	3964592	3,964592	785
eyflp; 73-79	*hsflp; 73-79*	**73-79**	73D1	79A4	123095	126201	16800857	21935345	5134488	5,134488	970
eyflp; 71-77	*hsflp; 71-77*	**71-77**	71B1	77C1	123418	125973	15007510	20394712	5387202	5,387202	999
eyflp; 70-77	*hsflp; 70-77*	**70-77**	70C6	77C1	124054	125973	13932268	20394712	6462444	6,462444	1131
eyflp; 70-78	*hsflp; 70-78*	**70-78**	70C6	78D5	124054	125886	13932268	21526856	7594588	7,594588	1371
eyflp; 70-79	*hsflp; 70-79*	**70-79**	70C6	79A4	124054	126201	13932268	21935345	8003077	8,003077	1467
eyflp; 70-80	*hsflp; 70-80*	**70-80**	70C6	80C2	124054	123975	13932268	22991401	9059133	9,059133	1725

Table S2. List of RS (Rearrangement Screening) FRT combinations in cis used in this work (related to Figure 2, 3)

List of recombinant fly lines bearing 21 different combinations of RS5r and RS3r-FRTs located in the same chromosome (*in cis*), where RS3r is distal to RS5r on the 3L (RS5r RS3r). Source of FLP expression, genomic location of RS-FRTs (cytogenetic and molecular), distance between RS-FRTs (in bp, Mb, and number of genes) are indicated.

FRT type	Line ID	Origin	insertion	orientation respect to chr
TSG GR	43615 (MiMIC)	BSDC	69F1	+
TSG RG	36936 (MiMIC)	BSDC	70A8	+
TSG GR	36936 (MiMIC)	BSDC	70A8	+
TSG RG	40163 (MiMIC)	BSDC	72A1	+
TSG RG	36406 (MiMIC)	BSDC	73A5	+
TSG GR	36406 (MiMIC)	BSDC	73A5	+
TSG RG	56571 (MiMIC)	BSDC	75A1	+
TSG GR	56571 (MiMIC)	BSDC	75A1	+
TSG GR	43044 (MiMIC)	BSDC	75F1	+
TSG RG	35938 (MiMIC)	BSDC	76A3	+
TSG RG	Griffin et al 2009	Perrimon lab	77C4	+
TSG GR	Griffin et al 2009	Perrimon lab	77C4	+
TSG GR	31404 (MiMIC)	BSDC	79A4	+
TSG GR	58655 (MiMIC)	BSDC	80B1	+
TSG GR	53833 (MiMIC)	BSDC	87A4	+
TSG RG	53833 (MiMIC)	BSDC	87A4	+
TSG GR	44927 (MiMIC)	BSDC	89A1	+
TSG GR	44869 (MiMIC)	BSDC	92F6	+

Table S3. List of TSG (Twin Spot Generator) FRTs used in this work (related to Figure 4-7)

TSG-FRTs (RG or GR) were injected into the indicated MiMIC lines. Line ID and origin (BSDC, Bloomington Drosophila Stock Center), genomic location and orientation of the insertion with respect to the chromosome are also indicated.
